# Organic Pig Farming in Europe: Pathways, Performance, and the United Nations Sustainable Development Goals (SDGs) Agenda

**DOI:** 10.3390/ani16030384

**Published:** 2026-01-26

**Authors:** Vasileios G. Papatsiros, Konstantina Kamvysi, Lampros Fotos, Nikolaos Tsekouras, Eleftherios Meletis, Maria Spilioti, Dimitrios Gougoulis, Terpsichori Trachalaki, Anastasia Tsatsa, Georgios I. Papakonstantinou

**Affiliations:** 1 Clinic of Medicine, Faculty of Veterinary Science, University of Thessaly, 43100 Karditsa, Greecegeopapak@vet.uth.gr (G.I.P.); 2 School of Economics, Aristotle University of Thessaloniki, 54124 Thessaloniki, Greece; 3 Department of Agricultural Economics and Rural Development, Agricultural University of Athens, 11855 Athens, Greece

**Keywords:** EU, organic, pig, sustainable development goals, SDGs, UN

## Abstract

Organic pig farming in Europe is endorsed as a promising route to more sustainable livestock production, but its ultimate contribution to the United Nations (UN) Sustainable Development Goals (SDGs) is a contested matter. This study takes a critical perspective on the potential of organic pig farming to contribute to SDGs that may include SDG 2 (Zero Hunger), SDG 3 (Good Health and Well-being), SDG 8 (Decent Work and Economic Growth), SDG 12 (Responsible Consumption and Production), SDG 13 (Climate Action), and SDG 15 (Life on Land). Organic farming systems delivered better animal welfare outcomes and positive benefits for biodiversity, soil health, and rural employment. Continued improvements in sourcing feed, greenhouse gas emissions per unit of product, animal health, and market could improve their contributions to agricultural sustainability. This study concludes that organic pig farming does not represent a guarantee of sustainable livestock production, but it could represent credible sources of sustainable livestock innovation if sufficient policy, practice, cost accounting, and sustainable metrics are organized together to support organic systems. Organic pig farming focused on innovation and policy support can make it a role model for the transition of European livestock sector towards the 2030 Agenda.

## Simple Summary

Organic pig farming could be an alternative for more sustainable livestock production, but its interaction with the United Nations Sustainable Development Goals (SDGs) remains debated. Present analysis takes a critical perspective on the potential of organic pig farming systems to contribute to SDGs that may include SDG 2 (Zero Hunger), SDG 3 (Good Health and Well-being), SDG 8 (Decent Work and Economic Growth), SDG 12 (Responsible Consumption and Production), SDG 13 (Climate Action), SDG 14 (Life Below Water), and SDG 15 (Life on Land). Drawing on recent studies, we assess the environmental performance, animal health and welfare outcomes, economic sustainability, and social context of pig farming in Europe. The study concludes that organic pig farming alone does not guarantee sustainability, but its practices can inspire innovation when backed by clear policies, practical measures, accurate cost analysis, and robust sustainability indicators. One does not guarantee sustainable livestock production; however, specific elements of organic systems may serve as valuable sources of innovation when reinforced by coherent policies, practical measures, accurate cost accounting, and robust sustainability indicators. Organic pig farming, while not inherently sustainable, has the potential to become a model for the European livestock sector’s transition toward the 2030 Agenda.

## 1. Introduction

Raising livestock, especially pigs, is a fundamental part of European farming, playing a vital role in providing food, jobs in rural areas, and international commerce. To illustrate, a recent study focusing on the EU-27 countries indicates that pig meat production in the EU reached approximately 23 million tons in 2022, representing about 21% of the world’s total [1]. It contributed 9.5% in 2020 to the total EU-27 agricultural output, the highest share within the meat sector [2]. The EU is the world’s top exporter of pig meat, with China as the main destination [3]. At the same time, there is rising societal expectation and policy impetus for livestock systems to become more sustainable: lower greenhouse gas emissions, reduced nutrient loss, improved animal welfare, and more circular resource flows. Sustainability has become a central goal for pig farming [4], as well as for agriculture in general [5,6]. It was added as one of the key objectives of the EU in the Amsterdam Treaty and was strengthened in 2001 by the publication of the EU Sustainable Development Strategy [7]. Sustainability is often defined as a “development that meets the needs of the present without compromising the ability of future generations to meet their own needs” [8].

The global food system is a main driver of climate change, contributing to approximately a third of total anthropogenic greenhouse gas emissions (GHG) [9,10,11]. Pig farming in Europe faces several sustainability challenges. The environmental impacts of pig production are widely criticized. Globally, pig farming accounts for an estimated 668 megatons of CO_2_-equivalent emissions each year, representing about 9% of livestock-related emissions [12]. Feed production, manure management (including storage and spreading), and land-use changes associated with soy cultivation contribute significantly to these environmental pressures [13]. Dramatic changes are needed in food production systems and consumption pattern needs urgent action to reduce GHG in ways that support resilience and sustainability, in line with the ambitions of the Paris Agreement, with a particular focus on strategies for scaling food systems with low GHG [14].

In 2015, the United Nations (UN) set out the 17 Sustainable Development Goals (SDGs) [15], a global strategy “to guide our common future in a new, better, and more deliberate way” [16]. Organic pig farming serves as a pertinent case study for examining the operationalization of SDGs, as it inherently integrates environmental sustainability, animal welfare standards, and socio-economic considerations within agricultural production systems. Although the SDGs do not explicitly include animal welfare, it is implicitly inalienable to the SDGs. There is a need for a comprehensive animal welfare law, accompanied by species-specific regulations, to ensure broader protection [17]. Farm animal welfare is gaining prominence on both moral and policy agendas, as many industrial practices in animal agriculture are increasingly being challenged. At the same time, academic scholarship show heightened interest in the relationship between humans and animals, particularly in examining how biologically and socially constructed boundaries between them are defined, maintained, or blurred [18]. The “One Welfare” concept, analogously to the “One Health” concept, illustrates how animal welfare, human well-being, and environmental sustainability are linked [19]. Into the future, Buller et al. [20] highlight that animal welfare will be at the center of many interconnected challenges related to food security, socio-economic development, human well-being, and environmental conservation. The SDGs provide a global framework to guide such transitions. In the livestock sector, the interplay between food security, environmental limits, animal welfare, and rural livelihoods is acute. Organic farming—by design—seeks to integrate many of these dimensions (health of soil–plant–animal–human systems, reduced synthetic inputs, improved welfare) under the principle that agriculture should be “ecologically, socially and economically” sustainable [21,22]. Sustainable agriculture is also a central component of Agenda 2030 [20]. Several UN Sustainable Development Goals (SDGs) address agricultural issues, such as SDG 2 “Zero Hunger”, SDG 12 “Responsible Production and Consumption”, SDG 13 “Climate Action”, SDG 14 “Life below Water”, and SDG 15 “Life on Land”, as agriculture not only contributes to but also impacts these SDGs [23]. Consequently, the EU has clearly emphasized the need for sustainable agriculture by launching the Green Deal and the Farm-to-Fork strategy [24], which address several SDGs. These SDGs are also incorporated into sustainability assessment tools to evaluate progress towards these goals.

Over the past three decades, organic farming has expanded annually across EU member states, although growth rates vary between countries. In 2015, the total number of organically raised pigs reached 0.978 million, with Denmark, France, and Germany representing the largest producers. Despite this growth, organic pig farming still accounts for less than 1% of the overall pig sector in the EU [25]. In Greece, pig farming is one of the most important branches of industrial livestock production, providing around 25% of domestic meat output and achieving a self-sufficiency rate of approximately 25–35% [26].

This article focuses specifically on organic pig farming in Europe because pig farming systems present distinct challenges (feed demand, manure and nutrient management, high productivity pressure, welfare issues) and offer opportunities for change. The objectives of this study are: (i) to map organic pig farming systems to relevant SDGs; (ii) to review empirical evidence on their sustainability performance; (iii) to analyze the main challenges and trade-offs; and (iv) to propose strategic recommendations for aligning organic pig production with SDG objectives.

## 2. Organic Pig Farming and Relevant SDGs

Organic pig farming represents an agricultural approach that integrates animal welfare, ecological processes, and reduced chemical inputs. Examining the relationship between organic pig farming and the UN SDGs reveals a complex network of interactions, akin to mapping the neural pathways of a small but highly intricate system. The wider relevance of organic pig farming extends into the global sustainability agenda, since its principles intersect closely with the Sustainable Development Goals (SDGs). While all 17 SDGs may in some way connect to livestock systems, a subset is particularly salient for organic pig farming in Europe. A summary of selected SDGs is presented in Table 1.

## 3. Sustainability Performance of Organic Pig Farming Systems in Europe

### 3.1. Environmental Dimension

The Animal Husbandry Alliance of IFOAM (International Federation of Organic Agriculture Movements) has stressed the need to evaluate environmental impacts of livestock production at a system level, not just focus on individual products [33]. Several studies assess pig farms using environmental life cycle tools. For example, in a large-scale study of 63 European pig farms (13 breeding, 27 breeding-to-finishing, 23 finishing farms), a multi-criteria assessment tool covering economy, environment, social well-being, as well as animal health and welfare was reported [33]. A moderate performance in parameters such as biodiversity and pig comfort, good performance in water use, and the human–animal relationship were reported, but considerable variability was highlighted [30]. A related study combining Life Cycle Assessment (LCA) with Key Performance Indicators (KPIs) focusing on biodiversity reported that LCA results (global warming potential, fossil energy demand, acidification, eutrophication) varied greatly across farm types, and that biodiversity scores (via KPIs) were independent of LCA performance [34]. The practical implication is that improving one dimension (e.g., biodiversity) may not guarantee lower greenhouse gas or nutrient emissions, and vice versa.

From the perspective of organic pig production, there is evidence that organic pig farming systems require higher land use and feed demand per unit of pork produced (due to slower growth and lower stocking density), which may increase the environmental burden per kilogram of output unless compensated by other advantages (such as better resource cycles or local feed). For example, a review and handbook for organic pig production states that outdoor access and lower densities improve welfare and biodiversity, but often at the cost of productivity and feed conversion [35].

Additionally, feed sourcing remains a crucial issue. A recent study on feeding strategy in organic pig farming argues that the new EU organic regulation since 2022 increasingly demands 100% organic feed and encourages stronger links to soil and circularity, potentially reducing imported high-impact feed components and thus improving environmental metrics [36]. A review by Pexas and Kyriazakis [37] highlights feed production (especially soy imports) and manure management as the two largest hotspots in pig systems, emphasizing that improved feed sourcing and manure strategies are essential.

### 3.2. Animal Health and Welfare

Organic pig farming can be an environmentally friendly way of farming based on the well known IFOAM core principles of health, ecology, fairness, and care, which can potentially help achieve some SDGs [38,39]. Organic pig farming has also acknowledged difficult challenges and debates affecting livestock systems and value chains.

Organic pig farming typically imposes stricter welfare rules: outdoor access or large pens, guaranteed rooting and foraging, prohibition or strict limitation of routine antibiotic use, and promotion of more natural behavior. Organic pig farming systems have sound welfare objectives; however, they still face major challenges, particularly disease risk and especially parasitism, as stated by Edwards [40]. Specifically, outdoor access may increase disease and parasitism risk (e.g., helminths, outdoor pathogens) and expose pigs to climate and weather stress, particularly in Northern Europe. The outdoor pig farming systems show increased piglet mortality in some contexts. Parasitic and respiratory infections are common, and elevated piglet losses have been reported on organic farms, as noted by Papakonstantinou et al. [26]. Hence, managing these risks is of prime importance if welfare gains are to translate into overall sustainability, according to Früh [35].

### 3.3. Economic and Social Dimensions

Over the past few decades, the operations of European pig farmers have been shaped by increasingly strict EU legislation aimed at reducing air, soil, and water pollution, improving animal welfare, and ensuring the social and economic viability of pig farming [41]. In addition, the growing market orientation of the EU’s Common Agricultural Policy (CAP), increasing sectoral fragmentation, the declining influence of marketing agencies, and rising societal expectations for sustainable agriculture have encouraged many farmers to reconsider their development strategies. Therefore, farmland diversification has again come into prominence as a means of reducing market-related risks and improving organizational efficiencies in resource use [42].

Organic pig producers have developed numerous strategies to cope with economic, legislative, labor, and climate-related shocks. Indeed, individual strategies prevail depending on the pig farming system and can include pig housing, diversification, and self-sufficiency in feed production. In this respect, it has been possible to identify three strategies: an efficiency-based strategy, a nutrition substitution strategy, and a farm diversification strategy. For all these strategies, policy specifically needs to be tailored to enhance resilience among pig producers [43].

According to the European Commission’s “Organic Farming in the EU” brief, organic animal production remains a small share of the total EU animal production, less than approximately 1% in some countries. This suggests that it is still a niche market; thus, limiting scale and economic elasticity, as noted by Papakonstantinou et al. [26]. Economic viability for organic pig farms is determined through access to premium markets, value-added supply chains, cost control—considering that organic feed is usually more expensive—and good farm management. Socially, organic pig farms are often smaller, more diversified, closer to local consumer networks, and may contribute to rural employment and resilience, thus aligning with SDG 8.

Insights into consumers’ attribute priorities, willingness to pay, and the trade-offs they accept provide an important basis for developing marketing strategies that promote more sustainable pork products [44].

### 3.4. Trade-Offs and Variability

For organic pig farming, trade-offs are significant: (a) lower productivity or slower growth requires more land or feed per kilogram of output, resulting in a higher environmental burden per unit unless mitigated; (b) outdoor access and welfare improvements can increase disease risk and heat stress, potentially leading to welfare, environmental, and economic penalties if mismanaged [26]; and (c) imported feed (such as soy) undermines biodiversity and land-use goals in feed-origin regions (SDG 15) unless replaced with local or circular feed systems. Additionally, variability across farms is high [33,34].

Organic livestock farming is increasingly recognized as a pathway toward more sustainable food systems, offering potential benefits for animal welfare, environmental protection, and rural development. To effectively plan the transition to organic pig farming toward sustainability, a SWOT analysis can be employed. This strategic tool evaluates the sector’s strengths and weaknesses, which arise from the internal environment, alongside the opportunities and threats of the external environment. A summary of these factors is presented in Table 2.

## 4. Challenges, Trade-Offs, and Bottlenecks

With the wider concept of sustainability, dilemmas may be expected to arise that involve trade-offs [45]. This interdependence also intersects in the SDGs [46]. Improvements on one SDG may on occasion impede improvements on others, a dynamic mapped as “conflicts” by Nilsson et al. [47]. Their analysis flags tension between SDG 2 Zero Hunger—which by implication involves increased production of food—and several of the other development goals. The main point here is that attempts to increase food will run into several different sustainability goals. In considering organic pig farming adhering to the SDG agenda, the following significant barriers arise:

### 4.1. Feed and Protein Sourcing

Organic pig farming systems require high-quality feed, which often includes organically certified imported soy that is linked with deforestation or land-use change elsewhere. Unless domestic or local protein crops—e.g., legumes—are developed, feed sourcing may undermine SDG 15, Life on Land [33]. Feeding strategy as a lever for improvement in organic pig farming is highlighted in a recent paper. Compared to diets that merely meet the minimal requirements of organic standards, feeding strategies based primarily on local resources—with diets rich in fiber and omega-3 fatty acids and supplemented with forage—have been shown to improve multiple quality dimensions of pork from non-castrated male pigs [36].

### 4.2. Productivity vs. Environmental Footprint

While welfare and biodiversity may improve, the slower growth and lower densities typical of organic pig farming systems increase the feed conversion ratio and land use per kg of pork. Unless mitigated, environmental gains per unit of output may be lower than in some conventional systems [33,34]. Despite growing awareness of environmental issues, comprehensive assessments of climate impacts on the EU pig sector remain limited. Prioritizing the development of forecasting models that integrate technical, economic, and resilience factors is essential to estimate medium- and long-term effects. Future research should also consider a range of socio-economic scenarios to better capture the complexity inherent in the VUCA (Volatility, Uncertainty, Complexity, Ambiguity) framework [48,49].

### 4.3. Animal Health and Welfare Risks

Although sustainability is a multidimensional concept, animal welfare concerns in livestock production have, until relatively recently, only occasionally been a high-profile component of the sustainability debate. Such is the case when Keeling et al. [50] note that even though animal welfare is not an SDG as such, considering its interactions with the accepted SDGs highlights its crucial role for the actual functionality of attaining those goals. Outdoor or semi-outdoor systems, although superior for behavior, may lead to increased risks of parasitism, external disease vectors, and heat or cold stress. For instance, high piglet mortality and health problems in organic conditions under specific circumstances are seen as expected by Papakonstantinou et al. [26].

### 4.4. Economic Viability—Scaling Challenges: Implications for Sustainable Development

Organic pig farming remains a marginal sector in Europe, most of the time below 1% of the total pig production. According to Papakonstantinou et al. [26], its economic viability is put under huge pressure because of structural limitations, cost inefficiencies, and low consumer demand for organic pork at premium prices. Organic pig production must meet strict welfare and environmental standards, such as access to outdoors, organic feed, and no synthetic growth promoters. The higher standards increase the cost of production exceeding that in conventional systems. Producers cannot achieve economies of scale (reduction in the cost of each unit produced as production increases) or sufficiently reduce per unit due to a low volume of production. Likewise, investment in infrastructure and other necessary upgrades is limited. In the absence of a regular market premium to compensate for the added costs, or when coupled with significant government support, many farms remain financially insecure. This situation cannot continue if the sector is to be viable in the long term. In that event, societal and economic impacts would be significant, notably for rural communities relying on agricultural diversity and resilience. Following are the SDGs of the United Nations that this directly relates to: SDG 8 (Good Jobs and Economic Expansion) and SDG 1 (Poverty Elimination). As for SDG 8, organic pig farming in theory sustains rural employment through labor-intensive practices and value-added local food systems. When economic viability is fragile, however, employment is unstable or becomes informal or poorly remunerated, which contradicts the aim for inclusive and sustainable economic growth. As for SDG 1, smallholders and family farms transitioning into organic systems often expect higher incomes through value-added markets. However, these expectations may not materialize in the absence of sufficient demand, infrastructure, or institutional support, placing producers at risk of financial hardship and perpetuating rural poverty.

Therefore, although organic pig farming corresponds to many of the principles of sustainable agriculture, its current limited scale and fragile market dynamics create a structural barrier to realizing developmental potential. This calls for targeted policy interventions, education of consumers, and formation of producer cooperatives to enhance bargaining power through aggregation of supply with a view of stimulating innovation.

### 4.5. Manure and Nutrient Management

While organic and low-density livestock systems are promoted as environmentally friendly alternatives, they are not exempt from nitrogen-related environmental challenges. Improper management of manure and nutrient surpluses can lead to significant ammonia and nitrate emissions, potentially undermining the environmental benefits of these systems [35,51]. The source of elevated emissions in organic or outdoor systems often lies in manure management: solid manure (common in straw-litter or pasture-based systems) tends to have higher pH during storage and slower infiltration after land application, which increases volatilization of ammonia [52]. To mitigate these emissions, improved handling is essential. Measures such as separating solids from liquids, reducing crude protein in animal diets (to decrease nitrogen excretion), using covered storage or impermeable covers, acidifying manure, or applying manure via injection rather than surface spreading, have been demonstrated to reduce ammonia emissions substantially [53].

### 4.6. Measurement and Standardization

Methods for the comprehensive assessment of all aspects of sustainability—environmental impact, human well-being, economic viability, and societal effects—are still largely under development. While progress has been made in creating frameworks and multivariable assessment tools that attempt to integrate these diverse dimensions, current approaches still face limitations [33]. For instance, some tools may omit important factors, rely on incomplete datasets, or struggle to quantify qualitative aspects such as social well-being or cultural impacts. Nevertheless, these assessment tools provide valuable insights and a structured basis for evaluating complex systems, guiding both research and policy decisions. Continued refinement and validation of these tools are essential to ensure more accurate, holistic, and actionable sustainability assessments in future studies [33].

### 4.7. Regulatory and Certification Constraints

The organic approval regulatory system is particularly stringent and constitutes one of the main barriers to the wider diffusion of organic pig farming in Europe. The EU organic rules, such as Regulation (EU) 2018/848 [24] and related legislation, require a high level of animal welfare, specific feeding regimes, appropriate housing conditions, and environmental protection. For pig farms, the exclusive use of organic feed and the requirement for daily outdoor access are among the most demanding standards, both posing significant practical and financial challenges [35,51].

One of the major challenges is the requirement for 100% organic feed, meaning that organic grains and protein sources must be used, which are generally more expensive and harder to obtain than conventional feed ingredients [53]. Most regions experience seasonal fluctuations in the availability of organic feed, which often leads to imports from other regions. This exposes producers to price volatility and supply-chain disruptions. Locally available high-protein organic feed is often in short supply, further increasing costs and environmental impact due to long-distance transport. This directly affects the feed conversion ratio—the efficiency with which animals convert feed into growth—and can significantly reduce profit margins, potentially threatening the economic viability of small and medium-sized farms striving to meet organic standards [53].

Similarly, the requirement for regular outdoor access—central to the organic principles of natural behavior and animal welfare—presents a major challenge for conventional farms converting to organic production. Most existing pig farms, particularly those built for intensive indoor systems, lack the land, fencing, shelters, or drainage necessary for appropriate outdoor areas [26]. This is especially relevant in regions without a tradition of pasture-based pig farming. Converting existing buildings or purchasing additional land to meet organic requirements involves high investment and attention to detail, which may discourage farmers from pursuing this transition. Furthermore, specific climatic conditions in parts of Europe make outdoor systems less desirable or less productive year-round, due to heavy rainfall or extreme temperatures. These factors also introduce additional animal health and biosecurity risks, which must be managed carefully [26].

Organic certification entails rigorous recordkeeping, regular inspections, and clear separation between organic and non-organic materials, particularly during the conversion period, which typically lasts one to two years for livestock operations. During this time, farmers must fully comply with organic standards without yet being able to market their products as organic, exposing them to financial risk and cash flow problems. This transition phase is one of the most vulnerable periods for organic farms, during which many farmers abandon the process due to a lack of support. While this regulatory and certification framework is designed to ensure the integrity of organic labeling and build consumer trust in organic food products, it has also created a substantial barrier for conventional pig farmers seeking to enter the organic market [54].

However, it must also be acknowledged that the very high standards and requirements imposed by these regulations are precisely what may contribute to the positive outcomes associated with organic farming—such as improved animal welfare, biodiversity protection, and consumer trust. These are not merely obstacles but the structural foundations of organic farming’s credibility. At the same time, it is important to clarify that organic standards represent only one part of the broader concept of sustainability and are not synonymous with it. Compliance with the regulations of organic pig farming can indeed serve as a meaningful step toward more sustainable practices, but this does not mean that everything labeled as organic is inherently sustainable in all respects [55].

## 5. Strategic Pathways and Recommendations

Organic pig farming in Europe has significant potential to advance the SDGs through environmentally responsible, welfare-friendly, and socially beneficial practices. Achieving this requires coordinated policy support, including financial incentives, harmonized regulations, and stakeholder engagement to promote sustainable production and local feed systems. Research and innovation in breeding, precision feeding, nutrient cycling, and welfare-oriented housing are essential to enhance productivity, resilience, and environmental performance. At the farm level, improvements in biosecurity, manure management, and precision livestock farming (PLF) technologies enable proactive health monitoring, data-driven decision-making, and reduced environmental impact. Market engagement through clear labeling, consumer education, and shorter supply chains strengthens demand for sustainable organic pork and supports local economies. Monitoring, reporting, and benchmarking using standardized sustainability indicators and GHG accounting frameworks further enable evidence-based management and alignment with carbon-neutral livestock strategies. Integrating these approaches positions European organic pig farming as a resilient, sustainable system contributing to SDGs across climate action, responsible consumption, animal welfare, and rural development.

### 5.1. Policy and Governance

To fully express the potential of organic farming, well-focused government policy support is highly necessary, together with finances and training for farmers. One more important aspect of transitioning towards organic farming is that all stakeholders, including farmers, consumers, officials, and scientists, will have to adopt a changed mindset and behavior [56].

Government support is crucial to meet the growing consumer demand for organic pig farms in Europe and to enable these farms to play a meaningful role in achieving international goals, including the SDG. In this regard, targeted financial support, supportive labeling, and accompanying programs, aligned with the EU’s strategic outlook on organic farming and organic food production, can offer the essential support needed. For example, organic farmland in the EU increased significantly between 2015 and 2020 (41%), but organic animal husbandry remains a very small share of the industry [41]. Promoting local, self-sufficient food systems centered on crops like beans and native grains represents an initial step in reducing the EU’s reliance on environmentally harmful imported feed, supporting both responsible consumption (SDG 12) and environmental protection (SDG 15). Linking manure management with anaerobic digestion and incentives for renewable energy can make manure from waste into a valuable product, both as a fertilizer and a source of energy, stimulating the circular economy and tackling climate change (SDG 13) [57]. Lastly, full harmonization of certification rules and regulations across countries will reduce paperwork, improve efficiency, and enhance compliance with organic standards by everyone, which should improve the sustainability of organic pig farming in Europe.

### 5.2. Research and Innovation

Advancing organic pig farming in Europe also requires targeted research and innovation. Breeding and pig genetics programs should focus on developing breeds or crossbreeds optimized for organic and outdoor systems, emphasizing slower growth with improved feed conversion and resilience to disease in semi-outdoor conditions. Precision feeding strategies and nutrient cycling approaches are essential to reduce nitrogen and phosphorus losses, improve manure management, and integrate pig-crop systems, such as pig-forage rotations, to close nutrient loops—needs highlighted in recent integrative environmental assessments [34]. Welfare-friendly housing and outdoor systems must be designed to mitigate heat and cold stress, reduce parasite and disease risks, and maintain behavioral freedom through access to outdoor rooting, straw bedding, and enrichment; numerous European best-practice approaches are summarized in the organic pig production handbook [35]. Finally, the development of harmonized, multi-dimensional sustainability assessment tools tailored to organic pig farms—including environmental, welfare, disease resilience, social, and economic indicators—is critical to accurately evaluate performance and guide improvements [36].

### 5.3. Farm-Level Practice Improvements

The farm needs several important elements to be improved if organic pig farming is to be in a better position to be sustainable: productivity, animal health, nutrient management, and product marketing. Increasing the efficiency of production factors such as feed use and reproduction will help decrease the environmental impact per unit of pork produced. There should also be strong levels of biosecurity and health management, specifically developed for outdoor and organic conditions; this will help prevent diseases and parasites from causing increased costs and resource use. A systematic risk-based approach, based on principles such as HACCP, can be used in managing such risks effectively. This view is supported by Papakonstantinou et al. [26] and Edwards et al. [40]. Proper handling of the manure, regarding storage, treatment, and application, is also warranted. Proper manure management in crop production allows recycling of nutrients as fertilizer, reducing synthetic fertilizers and moving toward a more circular economy. Lastly, chain improvement in traceability of pork and its marketing as “sustainable organic pork”, emphasizing animal welfare, environmentally friendly feed, and local supply chains, will attract consumers willing to pay a premium to offset the higher organic production costs.

PLF systems are revolutionizing modern, sustainable agriculture. PLF technologies could be useful tools for the farmers to enhance efficiency, reduce environmental impact, safeguard livelihoods, and improve animal health and welfare [58]. New technologies for gathering and analyzing data provide the foundation for structured farm management and decision-making. However, this approach requires the active engagement of all participants in the production chain [58,59]. Adopting PLF in organic systems requires cooperation among diverse stakeholders. Farmers and organic farm managers are central to implementing and operationalizing PLF technologies on the ground. Research institutions and universities contribute to validating and adapting tools to the specific conditions of organic farms. Certification bodies and control authorities must assess whether PLF use aligns with organic standards and principles [60,61]. Several barriers hinder PLF adoption in organic livestock systems. High investment costs for sensors, devices, and data infrastructure create economic challenges, especially for smaller farms. These costs are compounded by the need for training and technical support to use and interpret PLF data effectively. Technical challenges include interoperability between different PLF systems, validation of technologies under variable farm conditions, and the complexity of managing large volumes of data. There is also concern that technology may inadvertently diminish key aspects of traditional stockmanship or be perceived as incompatible with organic, low-input practices. Regulatory uncertainty about how PLF fits within organic certification frameworks can further slow adoption, and social factors such as farmer age, trust in technology, and resistance to change play a significant role [58,62,63]. PLF can support several SDGs at once, such as: (a) SDG 12 (Responsible Consumption and Production) and SDG 13 (Climate Action), when it reduces feed waste, antimicrobial use, and GHG; (b) SDG 3 (Good Health and Well-Being), when it improves animal welfare through early detection of pain, stress, or disease; and (c) SDG 2 (Zero Hunger), when it boosts farm efficiency and resilience.

### 5.4. Digital Innovation Pathways

Organic farming principles rely on maintaining robust animal health with minimal pharmaceutical intervention, which makes timely detection of health or welfare deviations particularly critical. PLF systems involve continuous monitoring, real-time temperature or activity sensors, automated body-condition scoring, environmental microclimate tracking, and data-driven feeding systems to allow farms to shift from reactive to proactive management [58]. These technologies enable early identification of stressors, parasitic burdens, nutritional imbalances, heat-stress events, or emerging disease risks before clinical symptoms develop, thereby reducing morbidity and mortality while maintaining compliance with organic standards that restrict routine antibiotic use.

Personalized health analytics, integrating longitudinal data from individual animals with farm-level environmental and nutritional records, support tailored interventions that optimize welfare while minimizing inputs. Combined with machine-learning models and decision-support dashboards, farms can benchmark health performance, detect anomalies in real time, and prioritize interventions aligned with One Health principles. Embedded early-warning tools can also facilitate better manure and nutrient management planning, predict heat-stress windows linked to climate change, and support long-term resilience modeling.

Integrating PLF-based personalized medicine approaches into organic pig farming strengthens several SDGs, including SDG 3 (Good Health and Well-being), SDG 12 (Responsible Consumption and Production), and SDG 13 (Climate Action). These technologies also promote transparency and traceability, improving consumer trust in organic pork value chains. Importantly, the adoption of early-warning systems should be supported by tailored training, advisory services, and incentives, ensuring equitable access for small and medium-sized organic farms. Digital monitoring tools represent a strategic opportunity to transition organic pig farming toward more resilient, data-driven, and welfare-enhancing systems [64].

### 5.5. Market and Consumer Engagement

If organic pig farming is going to fully realize its potential for long-term sustainability, more attention needs to focus on the market and what consumers want [65]. We can establish stronger systems for obtaining products to consumers by building recognizable brands, educating people about the advantages that organic pig farming has for animal welfare, the environment, and local jobs through clear certifications and labels [66,67]. In this way, consumers would be encouraged toward spending more and stimulating the market, since awareness strongly influences willingness to pay for organic meat [68].

The fact that organic pig farming meets several areas of sustainability—such as a production method serving the animal’s welfare, environmental protection, and the creation of local jobs—needs to be communicated not only to consumers but also to stores beyond the simple “organic” label [69]. This allows prices to reflect this extra value from good animal care and responsible environmental practices, supporting local economies [70]. Promoting shorter and more local supply chains and processing supports this approach, reducing the environmental impact of transport and enhancing links to local communities. Such regionalized food systems contribute directly to rural development and align with the attainment of decent work and economic growth (SDG 8) [46].

### 5.6. Monitoring, Reporting, and Benchmarking

**Figure 1:**
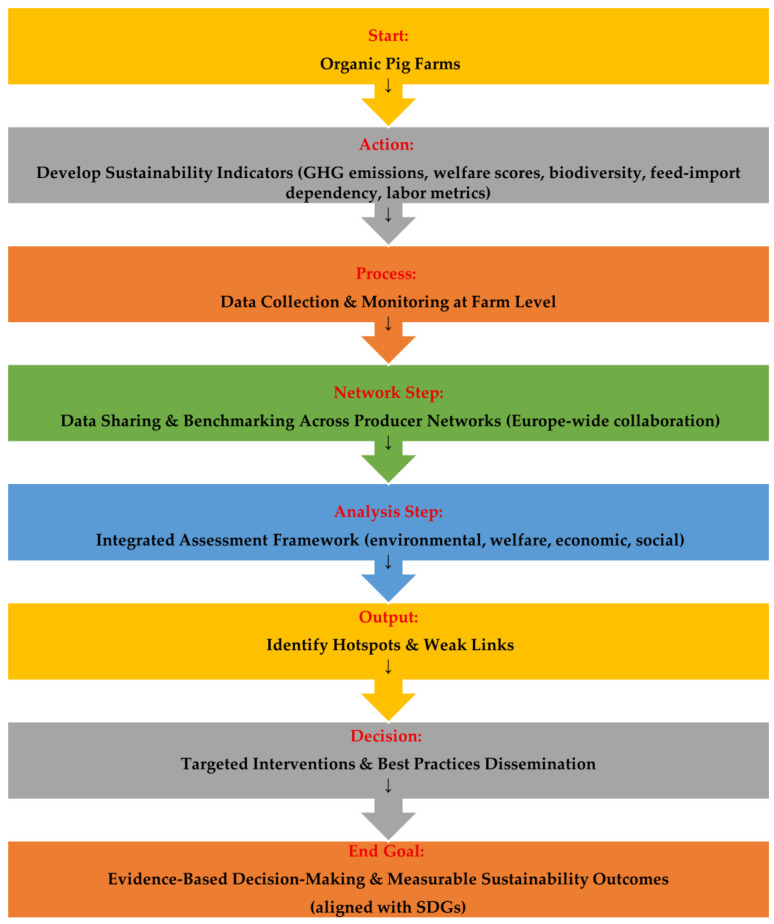
Evidence-based decision-making framework for sustainability monitoring, reporting, and benchmarking.

Effective monitoring, reporting, and benchmarking are essential to ensure that organic pig farms deliver measurable sustainability outcomes. The development of farm-level sustainability indicators for the SDGs—for example, a measure of emissions of greenhouse gases by kilogram of pork, welfare scores, biodiversity measures, feed-import dependency, and labor or employment by unit of output—creates the ability to systematically track performance. Networks of organic pig producers across Europe allow data exchange, benchmarking, and sharing of best practice, increasing collective learning and improvement. Integrated assessment frameworks, which combine environmental, welfare, economic, and social dimensions, allow for the identification of hotspots and weak links within farm systems, for instance, as described in Figure 1.

Within EU agricultural systems, organic farming has significant potential for reducing GHG emissions from agriculture, especially those related to crop production and livestock [71]. Major sources of agricultural GHG emissions are fertilization, plant protection, fossil fuel consumption for farm works, soil microbe activity, and animal emissions. Livestock production areas contribute significantly to GHG emissions under intensively managed production systems, including emissions along the livestock supply chain like feed production, enteric fermentation, and manure management. However, pasture-based production systems may reduce some emissions through carbon sequestration and the enhancement of biodiversity conservation [12,72,73,74]. Reductions in corresponding carbon footprints through the use of organic livestock production systems would also be realized by banning the use of synthetic chemicals, and mitigated reliance on external resources [75,76]. Extensive livestock systems can also help in reducing environmental pressure by sequestering more CO_2_ than is emitted through animal husbandry, which can contribute to GHG reduction [77]. These actions contribute to the larger objectives of sustainability through an emphasis on ecological balance and regenerative management of resources [69]. There is also a standardized way to assess sustainability, particularly GHG emissions, where the standard provides a structured process for quantifying, monitoring, and reporting emission GHG inventories, ensuring consistency, transparency, and accuracy in emissions accounting [78]. It is widely used in agriculture and livestock sectors, including organic pig farming, to measure carbon footprints and identify mitigation opportunities [77]. The ISO 14064-1:2018 standard includes principles for establishing GHG inventories, identifies direct and indirect sources of emissions, sets system boundaries for metrics, and encourages transparency in data collection and reporting [79]. Realistically and accurately understanding the environmental impacts associated with organic pig farming includes calculating its carbon footprint, which would include the emissions associated with enteric fermentation, manure management, feed production, land-use changes, and energy use along with the production cycle. The ISO 14064-1:2018 standard provides an accepted international method for the global quantification, reporting, and verification of GHG emissions at an organizational level, specifically for performance consistency and comparisons to other operations. The use of ISO 14064-1:2018, can help agricultural operations demonstrate metrics to account for policies under the European Green Deal and Farm-to-Fork Strategy based on evidence of mitigation and a transition to carbon-neutral approaches to sustainable livestock systems [12].

## 6. Discussion

Organic pig farming in Europe presents a nuanced pathway toward aligning livestock production with the United Nations SDGs, but its potential is closely tied to management practices, policy support, and systemic integration. The contribution of organic pig systems stems from their holistic qualities integrating welfare, environmental, and rural social aspects. The term *holistic* is critical here: focusing on a single dimension (e.g., welfare) without consideration of environmental or economic costs could undermine the overall sustainable outcomes of the system.

Organic pig farming systems in Europe raise significant questions to acquire SDG-aligned outcomes. Scale and productivity are key: most organic farms in the EU are smaller-scale farms with lower output per animal or hectare implying higher environmental costs per unit unless management and/or complementary practices sufficiently offset them. The design of the feed chain is equally important; to achieve SDG 13 (Climate Action) and SDG 15 (Life on Land) the reliance on imported feeds which often have higher impacts needs to be reduced and local/circular feed systems increased in the feed chain. From an environmental perspective, organic systems offer tangible benefits for biodiversity, soil health, and reduced chemical inputs, supporting SDG 13 (Climate Action) and SDG 15 (Life on Land). Carbon sequestration through pasture-based management, coupled with responsible manure and nutrient management, can partially offset the higher per-unit environmental footprint associated with slower growth and lower stocking densities. Feed sourcing is a critical leverage point: reliance on imported high-impact feed, such as soy, can undermine land-use and biodiversity goals unless replaced with local or circular feed systems. Integration of PLF technologies, including early-warning health systems and data-driven nutrient management, further enhances efficiency, reduces greenhouse gas emissions, and contributes simultaneously to SDG 12 (Responsible Consumption and Production). Animal welfare remains a core strength of organic pig farming, directly supporting SDG 3 (Good Health and Well-being). Outdoor access, natural behaviors, and reduced antibiotic use enhance animal health, while PLF-enabled monitoring allows early detection of stress, disease, or nutritional imbalances. These welfare improvements have indirect human health benefits by reducing antimicrobial resistance risk and promoting safer, more ethically produced food. On the socio-economic dimension, organic pig farming often operates at smaller scales, creating rural employment and strengthening local food chains, aligning with SDG 8 (Decent Work and Economic Growth). However, fragile economic viability, high production costs, and limited market scale constrain these benefits. Smallholders transitioning to organic systems may face financial risk without sufficient market demand, infrastructure, or institutional support, highlighting the need for targeted policies, cooperatives, and financial incentives.

Trade-offs remain a central challenge. While welfare and environmental quality can improve, slower growth rates and higher feed conversion ratios increase per-unit environmental costs unless offset by systemic improvements. Disease risk from outdoor systems and seasonal variations in feed availability also require proactive management to maintain welfare and productivity gains. These trade-offs illustrate that organic pig farming alone is not a guarantee of sustainable livestock production but can serve as a model for innovation when supported by integrated policies, monitoring frameworks, and sustainability-focused farm management.

Strategic pathways for maximizing SDG contributions include optimizing feed sourcing through local or circular systems, integrating precision livestock and digital monitoring technologies, strengthening rural market linkages, and harmonizing certification and reporting standards. Evidence-based sustainability monitoring, including GHG accounting under ISO 14064-1:2018, allows measurable contributions to SDG 2 (Zero Hunger), SDG 12, SDG 13, and SDG 15, translating global sustainability goals into actionable farm-level outcomes.

Systemic measures are also important; without sufficient tools that inform a broader multi-dimensional approach covering environment, welfare, heat economy, and social aspects, claims for organic systems to be sustainable will always be deficient [30]. Moreover, contextual factors such as climate, local market conditions, national subsidy regimes, farm size, and landscape characteristics all influence the effectiveness and sustainability of organic pig systems. Finally, policy and government support are crucial. Because organic pig farming makes up such a small part of the overall industry in many European countries—often less than 1% of production—targeted programs are needed to overcome the practical and financial barriers that currently limit the development of organic pig farming. This support would facilitate the growth of organic pig farming, enhance sustainability practices and enable a more meaningful contribution to several SDG objectives [38].

In summary, organic pig farming in Europe holds significant potential to contribute to multiple SDGs, particularly SDG 2, 3, 12, 13, and 15, with important social-economic co-benefits under SDG 8. Achieving these outcomes requires systemic management, policy support, innovation adoption, and rigorous monitoring. By addressing its current scale limitations, trade-offs, and structural barriers, organic pig farming could evolve from a niche sector into a resilient, environmentally responsible, welfare-focused, and socially beneficial component of Europe’s livestock systems.

## 7. Conclusions

While organic pig farming in Europe will not solve global problems on its own, it does represent a promising pathway toward aligning pig production with the globally agreed goals of a better future. Specifically, organic pig farming contributes positively to vital areas such as food availability, animal welfare, environmental care, and maintaining rural livelihoods. To realize the full potential of organic pig farming, however, some key challenges need to be overcome: notably enhancing resource use efficiency and productivity of farms, rethinking the feed production chain, reducing health and welfare hazards linked to outdoor rearing, ensuring a fair income for farmers, and establishing effective performance-tracking mechanisms. Overcoming these challenges opens the path for organic pig farming to go beyond its current niche role and to become an inspiring example of truly sustainable livestock production. The global goals for Agenda 2030 should therefore guide further development; organic pig farming may contribute substantially if it is further optimized.

SDGs are aligned with the United Nations Agenda 2030, particularly SDG 2 (Zero Hunger), SDG 12 (Responsible Consumption and Production), SDG 13 (Climate Action), and SDG 15 (Life on Land). By integrating organic pig farming systems with verified GHG accounting, it operationalizes Agenda 2030 at farm and value chain level, translating global sustainability goals into measurable, reportable, and verifiable outcomes. The approach demonstrates how livestock production can contribute to climate mitigation, biodiversity protection, and resilient rural livelihoods simultaneously.

ISO 14064-1:2018 plays a central role in underpinning the credibility of all environmental claims associated with the system. By providing a robust, internationally recognized framework for quantifying and reporting GHG emissions and removals, the standard ensures transparency, consistency, and comparability across farms and over time. Alignment with ISO 14064-1:2018 enables third-party verification, reduces the risk of greenwashing, and strengthens trust among policymakers, investors, supply-chain partners, and consumers. As such, the standard is not merely a technical add-on, but a foundational element for scaling organic pig farming as a credible climate-aligned solution.

Developing, engaging with, monitoring, and reporting on the environmental effects of organic pig farming, while keeping in mind the globally agreed goals, could pave the way for sustainable livestock production. Using established guidelines, such as ISO 14064-1:2018 for measuring GHG emissions, will lend credibility to these efforts. It would help farmers make verifiable and measurable claims about their environmental impact, enabling them to demonstrate that they are practicing organic yet still productive and animal-welfare-friendly farming, in addition to being seen contributing their part to help combat climate change. As global demand for protein increasingly looks toward more sustainable options, such as organic, coupling organic farming practices with environmental accounting frameworks seems a sensible approach for lessening the environmental impact of the livestock industry.

## Figures and Tables

**Table 1:** Connection and contribution of organic pig farming with selected SDGs.

SDG		References
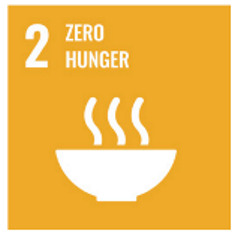	**SDG 2: Zero Hunger** Ensuring access to safe, nutritious food. Contributing diversified protein supply, supporting local value chains, and promoting food system resilience through less dependence on imported feed. Restricting routine antibiotics. Emphasizing soil health and nutrient cycling. Supporting long-term farm productivity and ecological resilience, as well as contributing to stable food production systems.	[27]
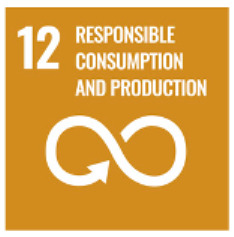	**Consumption and Production** Minimizing the use of synthetic fertilizers and pesticides, supporting nutrient circularity (between manure management), and emphasizing supply-chain traceability. Encouraging more circular nutrient flows, sustainable feed sourcing, and transparency in food systems.	[27,28]
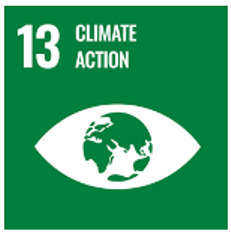	**Climate Action** Contributing to mitigation of GHG emissions. Enhancing carbon sequestration in comparison with conventional systems.	[29]
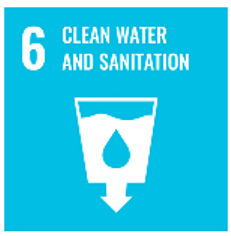	**and Sanitation** **Life Below Water** **15: Life on Land** Reducing water pollution by lowering the risk of excessive nutrient runoff and supporting practices that protect water quality. Protecting water-related ecosystems by minimizing chemical inputs and encouraging sustainable landscape features that buffer waterways. Mitigating nutrient runoff, avoid sourcing feed that drives deforestation (such as imported soy). Contributing to mitigating biodiversity loss in agroecosystems. Reducing nitrogen and phosphorus runoff, decreasing eutrophication in aquatic systems.	[28,30]
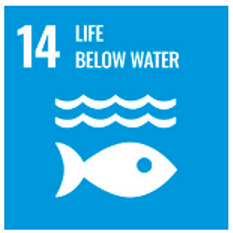		
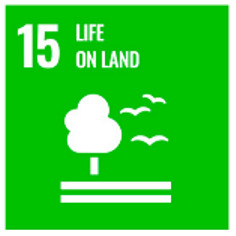		
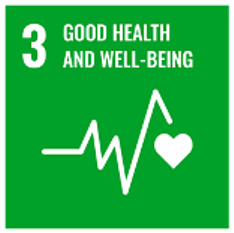	**and Well-being** Reducing stress on the animals. Reducing disease risk on the farm. Reducing antimicrobial resistance (AMR) potential risk.	[31]
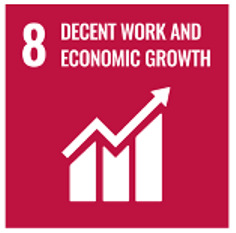	**Economic Growth** Associating with smaller-scale and more labor-intensive systems. Strengthening relationship with local supply chains. Creating rural employment and promoting equitable job opportunities.	[32]

**Table 2:** SWOT analysis for organic pig farming.

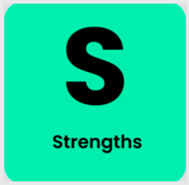	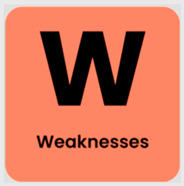
Ensuring high standards of animal welfare. Limiting antibiotics. Biodiversity conservation and water resources management. Supporting local communities. Growing consumer sustainability and responsibility. Promoting the use of 100% organic feed and fostering a circular economy through European regulations. Potential access to niche markets. Food self-sufficiency strategies.	Low productivity. High feed costs due to imported soybeans. Increased risk of diseases due to outdoor farming. Higher piglet mortality compared to conventional farming. Higher environmental footprint per product unit. Rising infrastructure demands and investments costs. Complicated and time-consuming certification process. High variability in yields among producers. Management problems associated with livestock manures and greenhouse gas emissions.
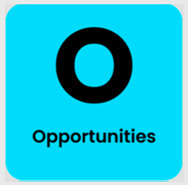	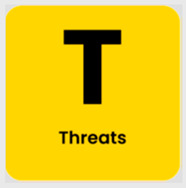
Development of local plant-based protein crops. Improved feeding strategies. Support policies for transition periods to ensure economic stability and creating cooperatives to increase bargaining power. Innovative solutions for better manure and greenhouse gas emissions. Opportunities for educating consumers about sustainable products. Development of forecasting tools for assessing climate impacts in the pig farming sector.	High production costs, which limit economic viability. Small scale of Europe’s organic pig farming sector hinders investment and innovation. Requirements for outdoor access for animals increase demands on land and infrastructure. Climate change. Heavy reliance on soy results in biodiversity loss Intense competition from conventional farms. Fragmented market and limited marketing activity. Challenges in reconciling well-being, productivity, and environmental goals.

## Data Availability

No new data were created or analyzed in this study.

## Abbreviations

The following abbreviations are used in this manuscript:

AMR	Antimicrobial Resistance
CAP	Common Agricultural Policy
EU	European Union Life Cycle Assessment
GHG	Greenhouse gas
HACCP	Hazard Analysis and Critical Control Points
IFOAM	International Federation of Organic Agriculture Movements
KPIs	Key Performance Indicators
LCA	Life Cycle Assessment
PLF	Precision livestock farming
SDGs	Sustainable Development Goals
SWOT	Strengths, Weaknesses, Opportunities, and Threats
VUCA	Volatility, Uncertainty, Complexity, Ambiguity
